# Secondhand Smoke Exposure and Maternal Action to Protect Children from Secondhand Smoke: Pre- and Post-Smokefree Legislation in Hong Kong

**DOI:** 10.1371/journal.pone.0105781

**Published:** 2014-08-28

**Authors:** Sophia Siu Chee Chan, Yee Tak Derek Cheung, Doris Yin Ping Leung, Yim Wah Mak, Gabriel M. Leung, Tai Hing Lam

**Affiliations:** 1 School of Nursing, The University of Hong Kong, Hong Kong, China; 2 School of Public Health, The University of Hong Kong, Hong Kong, China; 3 The Nethersole School of Nursing, The Chinese University of Hong Kong, Hong Kong, China; 4 School of Nursing, The Hong Kong Polytechnic University, Hong Kong, China; University of Texas at Tyler, United States of America

## Abstract

**Background:**

Smokefree legislation may protect children from secondhand smoke (SHS) in the home from smoking parent(s). We examined the effect of the 2007 smokefree legislation on children’s exposure to SHS in the home and maternal action to protect children from SHS exposure in Hong Kong.

**Methods:**

Families with a smoking father and a non-smoking mother were recruited from public clinics before (2005–2006, n = 333) and after the legislation (2007–2008, n = 742) which led to a major extension of smokefree places in Hong Kong. Main outcomes included children’s SHS exposure in the home, nicotine level in mothers’ and children’s hair and home environment, mothers’ action to protect children from SHS, and their support to the fathers to quit.

**Results:**

Fewer mothers post-legislation reported children’s SHS exposure in the home (87.2% versus 29.3%, p<0.01), which was consistent with their hair nicotine levels (0.36ng/mg versus 0.04ng/mg, p<0.01). More mothers post-legislation in the last month took their children away from cigarette smoke (6.3% versus 92.2%; p<0.01) and advised fathers to quit over 3 times (8.3% versus 33.8%; p<0.01). No significant change was found in the content of smoking cessation advice and the proportion of mothers who took specific action to support the fathers to quit.

**Conclusions:**

SHS exposure in the home decreased and maternal action to protect children from SHS increased after the 2007 smokefree legislation. Maternal support to fathers to quit showed moderate improvement. Cessation services for smokers and specific interventions for smoking families should be expanded together with smokefree legislation.

## Introduction

The prevalence of daily smoking in Hong Kong has declined from 23.3% in 1982 to 10.7% in 2012 [1]. Passive smoking killed 1324 people in 1998 in Hong Kong [2]. This figure was reported in advocacy campaigns to gain public support leading to the enactment of stronger smokefree legislation in October 2006, implemented from 1 January 2007. In the past two decades, smokefree legislation has been enacted or strengthened in some developed countries or regions to reduce secondhand smoke (SHS) exposure among non-smokers including children. By the end of 2007, 16 countries had passed national smokefree legislation covering all indoor workplaces and public sites [3]. Since 1 January 2007, Hong Kong extended the smokefree areas substantially, by banning smoking in all indoor workplaces, indoor areas of restaurants, shops, markets, public transport, residential care homes, indoor and outdoor areas of all educational institutions (including nurseries, kindergartens, schools and universities). Outdoor common areas such as public playgrounds, parks, beaches, barbecue sites, public swimming pools and all common areas of public housing estates (except a few outdoor smoking areas at remote corners) were also designated as smokefree areas.

Smokefree legislation is an effective intervention for smoking cessation. Previous studies found a reduction of smoking prevalence after the legislation in the United States, Canada, Italy and Scotland [4]–[6]. Smokefree legislation was associated with increased quit attempts in Scotland, England, and Ireland [7]–[10]. In Hong Kong, the 2007 smokefree legislation had a short-term effect in increasing the utilization of quitline services [11], [12].

Other studies have examined the two contrasting hypotheses for the impact of smokefree legislation: displacement effect and social diffusion of indoor smoking. Displacement effect means that the legislation reduces smoking in public, but simultaneously increases cigarette consumption in the homes and SHS exposure in other family members [13], [14]. Social diffusion denotes that increasing restriction of smoking in public places would raise public awareness on SHS, resulting in more home smoking restriction [15]. The findings about these two hypothesized effects were inconsistent. Displacement effect of smoking was shown in Hong Kong and the US [13], [16], but was not found in England [4], [17], [18], Wales [19], [20], Scotland [20]–[22] and some European countries [23]. Some studies found that post-legislation reduction of SHS exposure was limited to non-smoking families or families with only the fathers being smokers [21], [24]. Also, the SHS exposure in families with members smoking in the home showed no change between pre- and post-legislation [21], [24], [25].

Several studies above investigated the association between smokefree legislation and reduced SHS exposure in in-school adolescents and children aged above 4. Only one previous study specifically examined the impact of legislation on smoking behaviors of parents having children aged under 9 months [4]. Infants are vulnerable to respiratory illnesses from passive smoking as they have smaller and less developed lungs [26], [27]. 165,000 children younger than 5 years in the world die every year from lower respiratory infection caused by SHS exposure [28]. On the other hand, the implementation of smokefree legislation significantly reduced the hospital admissions for asthma in children [29], [30]. In Hong Kong, the prevalence of daily smoking of Hong Kong males and females was 19.1% and 3.1%, respectively [1]. Paternal smoking is the major source of SHS in the home and the risk factor for respiratory or febrile illness in infants [31]. Hence, the present study targeted families with a smoking father, a non-smoking mother and a child aged 18 months or below.

Moreover, the association between smokefree legislation and maternal action to protect children from SHS in the home has not been examined. The maternal action includes bringing children away from smoking, asking smokers not to smoke near children, or giving cessation advice to smokers. Our previous study in Hong Kong found that spousal advice and support can help the smoking spouse to quit [32]. However, Chinese familial value follows Confucianism, which shapes family relationship in an expectation of respect for elders and males, in order to maintain a patriarchal and harmonious family [33]. A married woman in a Confucian, albeit highly westernized, society is less likely to challenge her husband’s smoking habit [26], [33].

The present study aimed to (1) examine the change of the paternal smoking behavior and quitting, (2) examine the change of the children’s SHS exposure in the home, (3) examine the change of the mothers’ action to protect their children from SHS and help the fathers to quit smoking, and (4) investigate whether the displacement or the diffusion hypothesis was supported after the extension of smokefree legislation.

## Materials and Methods

### Study design

We conducted an analysis based on the 3 cross-sectional survey data collected pre- and post-smokefree legislation. The pre-legislation data was drawn from two pilot randomized controlled trials (RCTs) in 2005 and 2006 [34]–[36]. The post-legislation data was drawn from a cross-sectional survey in 2007 and 2008. To maximize comparability, the recruitment strategy and eligibility criteria for the post-legislation survey were the same as the RCTs in 2005 and 2006. Both parents in the 2005 RCT and the post-legislation survey, and fathers in the 2006 RCT were invited to complete a standardized questionnaire. In addition, SHS exposure was assessed via nicotine level in mothers’ and children’s hair, and the level in their homes measured with nicotine monitors in the 34 families in the RCT in 2006 and 34 demographically matched families (by 7 criteria, see below) in the post-legislation survey.

### Settings

Recruitment of all families was done in Maternal and Child Health Centres (MCHC) and Student Health Service Centres (SHSC) assigned by the Department of Health of the Hong Kong Government, as these centres had the largest number of clients for easier recruitment. MCHC provides free and integrated health services (e.g. vaccination and health check) for all newborn babies and all children up to the age of 5 who are Hong Kong residents. SHSC offers annual free health services to all students in primary and secondary schools including physical examination, individual health counselling and health education.

### Comparison of the groups

The pre-legislation group consisted of 2005 and 2006 RCT participants. The 2005 data were from a baseline survey of a pilot RCT to test a nurse-delivered smoking hygiene intervention for non-smoking mothers to reduce SHS exposure in the household [36]. 219 families (including both fathers and mothers) were recruited from two MCHCs in the two districts of Lamtin and Yaumatei from January to April 2005. All the children were aged 18 months or below.

The 2006 data were from a RCT to examine the impact of feedback on SHS exposure among non-smoking mothers and children in the home on the father’s smoking behavior [34], [35]. Of the 120 screened families in two MCHCs in the two districts of Hunghom and Wan Chai and two SHSCs in two other districts of Lamtin and Sai Wan from May to October 2006, 114 (95%) consented to participate and only the fathers were required to complete the questionnaire on smoking behavior.

The post-legislation data were obtained by conducting a cross-sectional survey on the parents recruited from the four MCHCs and two SHSCs, which were the same as the 2 RCTs, plus the SHSC in one more district of Shatin, from June 2007 to August 2008. 1857 of the 12, 011 (15.5%) screened families were eligible and invited to participate. 918 families (49.4%) consented and among them, 576 families (62.7%) with both parents, and 198 families (21.6%) with either one of the parents completed the survey. 144 families (15.7%) withdrew their consent. No participants in the survey reported participation in the 2005 and 2006 RCTs. In total, 742 mothers (80.8%) and 604 fathers (65.8%) completed the questionnaire.

Overall, 219 parents in the 2005 RCT, 114 fathers in the 2006 RCT, and 742 mothers and 604 fathers in the 2007–2008 survey were analyzed in the present study. For the comparison of fathers’ smoking and quitting between the pre- and post-legislation group, all father-reported data in the three surveys were used. For the comparison of children’s exposure to SHS and maternal action of protecting children from SHS exposure, only the mother-reported data from the MCHCs in 2005 RCT (n = 219) and 2007–2008 survey (n = 183, excluding those mothers with a child aged over 18 months) were included. Only 15 of the 144 children in the 2006 RCT were aged 6 years or below, so the data of 2006 RCT was excluded in the latter comparison. The comparison of nicotine level for children, mothers and the home environment was carried out with the data from the 2006 RCT and the post-legislation survey. The data is available upon request submitted to the corresponding author.

### Eligibility

The inclusion criteria for the 2005 and 2006 RCTs were (1) the non-smoking mother accompanying her child to attend the selected MCHC/SHSC, (2) the child was under 12 years old, (3) the child’s father was a current smoker who smoked at least 1 cigarette per day in the past 30 days, (4) the mother, father, and child lived in the same household for at least 5 days in the past week, (5) both parents spoke Cantonese, and (6) both parents were Hong Kong residents. In order to obtain comparable samples in the post-legislation survey, the inclusion criteria were the same as the two RCTs. For the two RCTs and the survey, families with smoking fathers undergoing other smoking cessation programmes were excluded.

### Procedures

For the two RCTs and the survey, parent(s) attending the selected MCHCs with their child on a selected date or students visiting the selected SHSCs with their parent(s) were invited to participate. Trained research assistants approached the families in the study sites and screened for their eligibility. After obtaining consent from both parents, the research staff administered a standardized and structured questionnaire for each participant at the study site. In case the father was absent at the study site, the research staff called him to obtain verbal consent and complete the survey via telephone within two days.

### Variables in the self-administered questionnaire

The main outcomes were father-reported daily cigarette consumption, Fagerstrom Score of Nicotine Dependence [37], situations of smoking, quit attempt experience, stage of change in the Transtheoretical Model of Change [38], mother-reported children’s exposure to SHS, and mothers’ action to protect children from SHS. Details are shown in Table S1 in File S1.

### Direct measurements of SHS exposure

SHS exposure was assessed by hair nicotine level of the mothers and children, and air nicotine level in the home. The procedures of collecting the test samples were parts of a multinational study of SHS exposure among women and children in 2006 [35]. Hair samples were collected in 34 families in the intervention group of the 2006 RCT and 34 demographically matched families in the post-legislation survey. Due to budget constraint in the 2006 RCT, only 34 of the 114 families could be included in the intervention group in which the nicotine level was measured. The cost of performing the nicotine measurement for all the participants in the post-legislation survey was high, and to ensure comparability between the subjects pre- and post-legislation, we used 7 criteria to match the newly recruited families in the post-legislation survey to the 34 families in the 2006 intervention group as follows: (1) study site, (2) age of child within 2 years, (3) age of father within 5 years, (4) age of mother within 5 years, (5) education level of father (3 groups: primary or no formal education, F1–F5, and above F5), (6) education level of mother (3 groups) and (7) cigarettes smoked per day by fathers (3 groups; 1–15 cigarettes, 16–25 cigarettes, above 25 cigarettes). The comparability was further assured such that the mean daily cigarette consumption of fathers in both groups were not significantly different (17.1 (pre) vs 15.3 (post), p-value = 0.46). The selected families were informed that one passive nicotine monitor would be placed for a week in the main room of their homes where the family congregated. Hair samples (approximately 30 strands) were taken from the children and the mothers on the day the passive air monitors were installed. The procedures for nicotine measurements were prescribed by the Johns Hopkins University project and were applied again in measuring the nicotine level in the post-legislation families [35]. In the study of Wipfli etal. (2008), blank and duplicated samples were collected to determine the limit of detection (i.e. reading of a blank sample from a non-smoking family) for quality control purpose. The median limit of detection was 0.001 ug/m^3^ for a 7-day air sample in the home, and 0.02 ng/mg for a 30-mg hair sample. Final nicotine concentrations of the samples were calculated by subtracting the median limit of detection from the actual readings.

### Statistical analysis

We pooled the pre- and post-legislation samples and set the time variable (pre- or post-legislation) as the explanatory variable in the multivariate regression models. The differences in the outcomes (listed in Table S1 in File S1) between the pre- and post-legislation group were tested with regression coefficients or odds ratios. To examine the effect of the legislation on the binary and ordinal outcomes, adjusted odds ratios for post- versus pre-legislation were obtained from binary logistic regression and ordinal logistic regression, respectively. The coefficient for the ordinal logistic regression represented the magnitude of change in log-odds of having worsen outcome for subjects in post- versus pre-legislation. Odds ratios from ordinal logistic regression less than 1 indicated the decreased likelihood of higher outcome (e.g. categories of more cigarette consumption) in subjects post-legislation compared with their counterparts pre-legislation. For continuous outcomes, multiple linear regression was used to determine the coefficients of the exposure for post-legislation, indicating the direction and magnitude of the post-legislation effect. The results from the regression models were adjusted for age (father, mother & child), father’s education level, years of father’s smoking, father’s perceived health status, children’s medical consultation in the past month, household income level and number of children in the home. Nicotine level in hair and air environment was summarized using median and range. Since Kolmogorov-Smirnov tests found that the distributions for the nicotine level were not normal, Mann-Whitney U non-parametric test was used to compare the hair and air nicotine levels between the 2006 RCT and the post-legislation group. SPSS 20.0 was used for all analysis.

### Ethics approval

All the consent procedures and data collections were approved by the Institutional Review Board of the University of Hong Kong and Hospital Authority Hong Kong West Cluster (Reference no.: UW 07-065). All parents in the RCTs and the survey gave written consent to the interview and the collection of saliva samples from mothers and children. In case the father was absent in the study site, we telephoned the father to obtain a verbal consent followed by the interview.

## Results

There were significant differences in the education level of both parents, household income, perceived physical health, and the number of children at home between the pre- and post-legislation group (See Tables S2 and S3 in File S1). These variables were adjusted in the multivariate analysis.

### Smoking habit and quitting (Father-reported)

Table 1 shows that the distribution of fathers’ daily cigarette consumption was similar between the pre- and post-legislation group, but there was a slight increase in the proportion of smoking 10 cigarettes or below (post: 38.4%, pre: 32%, p for the ordinal regression model = 0.01). Smoking in most situations, including in the home, was less frequently reported post-legislation; though the proportion reporting smoking when their children were not nearby increased significantly (post: 41%, pre: 22.4%, p<0.01). The differences in the smoking situations from the crude comparisons were further supported by the significant odds ratios after the adjustment of other covariates. The father’s Fagerstrom Score of Nicotine Dependence between pre- and post-legislation showed no significant difference when analyzed as ordinal categories (Adj. OR from ordinal regression model = 0.88, 95% CI 0.60–1.29), but the post-legislation group had a significantly lower mean score than the pre-legislation group (post:2.88, pre:2.96, p-value = 0.04).

**Table 1:** Father-reported smoking and quitting pre- and post-legislation.

	Pre-legislation2005–2006(n = 333)n(%)	Post-legislation2007–2008(n = 604)n(%)	Adjusted odds ratios(95% CI)/Regressioncoefficient	p-value for theadjusted odds ratios
Mean daily cigarette consumption in the past week			1.56(1.08, 2.24)	0.01
More than 30 cigarettes	9(2.8)	16(2.7)		
21–30 cigarettes	20(6.2)	47(8.0)		
11–20 cigarettes	189(58.7)	300(50.9)		
10 cigarettes or below	103(32.0)	226(38.4)		
Mean daily cigarette consumption whensmoked most heavily (SD)	23.4(11.7)	19.1 (13.9)	−4.87	<0.01
Fagerstrom Nicotine Dependence Test			0.88(0.60, 1.29)	0.52
Mild (Score 0–3)	203(63.0)	358(61.9)		
Moderate (Score 4–5)	82(25.5)	143(24.7)		
Severe (Score 6–10)	37(11.5)	77(13.3)		
Mean score (SD)	3.0(2.0)	2.9(2.2)	−0.39	0.04
Situations when smoked				
At home	211(65.5)	228(38.5)	0.18(0.12, 0.28)	<0.01
At work	233(72.4)	356(60.2)	0.35(0.23, 0.54)	<0.01
When relaxing	275(85.4)	324(54.7)	0.09(0.05, 0.17)	<0.01
When felt bored/want to kill time	275(85.4)	312(52.7)	0.07(0.03, 0.13)	<0.01
Wanted to increase concentration	60(18.6)	159(26.9)	1.95(1.20, 3.17)	0.01
Felt anxious	253(78.6)	182(30.7)	0.06(0.04, 0.10)	<0.01
In the absence of my children	72(22.4)	243(41.0)	2.77(1.76, 4.34)	<0.01
Smokers around	273(84.8)	265(44.8)	0.05(0.03, 0.10)	<0.01
After meal	289(89.8)	386(65.2)	0.05(0.02, 0.11)	<0.01
Drinking alcohol	148(46.0)	227(38.3)	0.78(0.53, 1.13)	0.11
**Quitting**				
Tried to reduce smoking	246(76.4)	471(78.0)	0.94(0.61, 1.45)	0.78
Had previous quit attempt	194(60.6)	363(61.7)	0.93(0.64, 1.36)	0.72
Stage of readiness to quit				
Pre-contemplation	304(94.4)	509(86.9)	3.74(1.93, 7.24)	<0.01
Contemplation	13(4.0)	40(6.8)		
Preparation	5(1.6)	8(1.4)		
Action	0(0.0)	29(4.9)		

Remark: For all regression models, odds ratios and regression coefficients were adjusted by age (father, mother & child), father’s education level, years of father’s smoking, father’s perceived health status, child’s consultation to doctor in the past month, household income level and number of children at home. Missing data were excluded from analysis.

There were no significant differences in reducing smoking and quit attempt between the pre- and post-legislation group, but the proportion at the action stage of quitting was higher post-legislation (post: 4.9%, pre: 0%, p-value<0.01).

### Mother-reported children’s exposure to SHS and nicotine level in hair and home environment

Table 2 shows that the hair nicotine level in mothers and children post-legislation was lower based on the Mann-Whitney U test. The air nicotine level in the home was low and nearly undetectable pre- and post-legislation, which is consistent with the finding that the proportions of father’s smoking at home between pre- and post-legislation were similar (pre: 94.2%, post: 79.4%, p = 0.07). Table 3 shows, as reported by mothers, more fathers post-legislation did not smoke at home (pre: 10.1%, post: 37.7%, p<0.01). 85.4% of fathers post-legislation did not smoke near the children, compared with 17.0% pre-legislation (p<0.01). 29.3% of children post-legislation were exposed to SHS in the home, compared with 87.2% pre-legislation (p<0.01). All the odds ratios in Table 3 were significantly smaller than 1, meaning that the odds of being in the worsen outcome categories in the post-legislation group were smaller than the pre-legislation group. Fathers post-legislation were less likely to smoke near children or in the home, and children were less likely to be exposed to SHS in the home or near other smokers.

**Table 2:** Nicotine level in mothers’ and children’s hair and home environment pre- and post-legislation.

Nicotine level	Pre-legislation2006(n = 34)	Post-legislation2007–2008(n = 34)	p-value for Mann-Whitney U test
Child’s hair in ng/mg, Median (Range)	0.36 (0.09–11.88)	0.04 (0.01–0.58)	<0.01
Mother’s hair in ng/mg, Median (Range)	0.29 (0.09–1.16)	0.03 (0.01–9.74)	<0.01
Air at home µg/m^3^, Median (Range)	0.01 (0.004–0.27)	0.01 (0.001–0.73)	0.58

Remark: Missing data were excluded from analysis.

**Table 3:** Mother-reported father’s smoking behavior at home and children’s exposure to SHS pre- and post-legislation.

	Pre-legislation 2005(n = 219)n(%)	Post-legislation 2007–2008 (n = 183)n(%)	Adjusted odds ratios (95% CI)	p-value for the adjusted odds ratios
Father’s cigarette consumption within10 feet of the child in the past week			0.05(0.02, 0.09)	<0.01
None	37(17.0)	152(85.4)		
Less than 1 cigarette per day	75(34.4)	10(5.6)		
1–4 cigarettes	83(38.1)	12(6.7)		
5–14 cigarettes	21(9.6)	3(1.7)		
More than 14 cigarettes	2(0.9)	1(0.6)		
Father’s daily cigarette consumptionat home in the past week			0.40(0.23, 0.70)	<0.01
None	22(10.1)	58(37.7)		
Less than 1 cigarette per day	31(14.2)	3(1.9)		
1–4 cigarettes	125(57.3)	69(44.8)		
5–14 cigarettes	38(17.4)	22(14.3)		
More than 14 cigarettes	2(0.9)	2(1.3)		
Number of smokers (excluding father)smoked within 10 feet of the child in thepast week			0.05(0.02, 0.10)	<0.01
0	67(30.6)	165(91.7)		
1	138(63.0)	13(7.2)		
2	12(5.5)	2(1.1)		
3 or above	2(0.9)	0(0.0)		
Child’s SHS exposure in the home			0.08(0.04, 0.14)	<0.01
No exposure	28(12.8)	128(70.7)		
Occasional	86(39.3)	36(19.9)		
1 hour per day	57(26.0)	11(6.1)		
2–4 hours per day	45(20.5)	3(1.7)		
5–7 hours per day	2(0.9)	2(1.1)		
8–10 hours per day	1(0.5)	1(0.6)		

Remark: Values are number (%). For all regression models, odds ratios were adjusted by age (father, mother & child), father’s education level, years of father smoking, father’s perceived health status, child’s consultation to doctor past month, household income level, and number of children at home. Missing data were excluded from analysis.

### Mother’s action to protect children from SHS

Table 4 shows that in mothers whose children were exposed to SHS, the mothers post-legislation were more likely to take their children away from SHS than pre-legislation (pre: 6.3%, post: 92.2%, p<0.01), placed a ‘No-Smoking’ sign at home (pre: 0.5%, post: 17.6%, p = 0.01) and advised the fathers to avoid smoking near their children (pre: 69.1%, post: 86.3%, p = 0.03). Over 90% of mothers pre- and post-legislation advised the fathers to reduce smoking, avoid smoking at home or avoid smoking near the children.

**Table 4:** Mother’s action in protecting the child from SHS exposure, and mother’s advice and support in helping father to quit pre- and post-legislation.

	Pre-legislation 2005n(%)	Post-legislation 2007–2008n(%)	Adjusted odds ratios(95% CI)	p-value for the adjusted odds ratios
**Protecting child from SHS exposure, among those mothers whose** **children were exposed to secondhand smoke**	(n = 191)	(n = 51)		
Took the child away from smoke	12(6.3)	47(92.2)	325.29(40.21, 2631.69)	<0.01
Opened the window	186(97.4)	44(86.3)	0.05(0.006, 0.43)	0.01
Placed a ‘No-Smoking’ sign at home	1(0.5)	9(17.6)	21.01(2.13, 207.54)	0.01
Advised father to reduce smoking	185(96.9)	47(92.2)	0.70(0.06, 8.81)	0.78
Advised father to avoid smoking at home	168(88.0)	45(88.2)	2.21(0.59, 8.21)	0.24
Advised father to avoid smoking near the child	132(69.1)	44(86.3)	3.68(1.15, 11.75)	0.03
**Number of mothers’ advice to the fathers to quit in past month,** **all mothers**	(n = 219)	(n = 183)	2.23(1.32, 3.78)	<0.01
Never	75(34.2)	65(35.9)		
Advised 1–3 times	126(57.5)	56(30.9)		
Advised 4–6 times	14(6.4)	20(11.0)		
Advised 7–9 times	3(1.4)	6(3.3)		
Advised more than 9 times	1(0.5)	34(18.8)		
**Content of smoking cessation advice, among those mothers who** **advised fathers to quit**	(n = 144)	(n = 116)		
Reminded him about the benefit to the child’s health	55(38.5)	63(54.3)	1.64(0.82, 3.30)	0.16
Reminded him that smoking can lead to death	37(25.9)	37(31.9)	1.31(0.62, 2.77)	0.49
Reminded him that quit smoking can save money	41(28.7)	32(27.6)	0.73(0.34, 1.56)	0.41
**Mothers’ action to help fathers quit, among those mothers** **who advised the fathers to quit**	(n = 144)	(n = 116)		
Any action done	33(23.1)	31(26.7)	1.22(0.56, 2.67)	0.62
Set a quit date for him	1(0.7)	5(4.3)	5.44(0.55, 53.53)	0.15
Removed all the smoking-related utensils	9(7.8)	9(6.3)	0.97(0.27, 3.45)	0.96
Placed a ‘no-smoking’ sign at home	1(0.7)	7(6.0)	6.79(0.51, 91.17)	0.15
Requested others not to smoke near the father	9(6.3)	5(4.3)	1.72(0.23, 12.83)	0.60
Gave father smoking cessation booklet	19(13.3)	8(6.9)	0.36(0.12, 1.14)	0.08
Advised to seek professional help	2(1.4)	9(7.8)	10.05(1.47, 68.60)	0.02
Discussed with father of needs in quitting	1(0.7)	10(8.6)	1.64(0.82, 3.30)	0.16
**Mothers’ support in helping fathers quit, among those who** **advised fathers to quit**	(n = 144)	(n = 116)		
Any support given	40(28.2)	36(31.9)	1.06(0.50, 2.24)	0.88
Complimented father when he did not smoke	11(7.7)	21(18.6)	3.55(1.17, 10.76)	0.02
Congratulated him for decided to quit	2(1.4)	4(3.5)	3.29(0.27, 39.47)	0.35
Helped father to think of substitutes for cigarettes	30(21.1)	7(6.2)	0.35(0.12, 1.05)	0.06
Comforted father when he was feeling stressed or irritated	9(6.3)	6(5.3)	0.64(0.14, 2.96)	0.57
Expressed confidence in father’s ability to quit/remain quitting	5(3.5)	2(1.8)	0.10(0.01, 1.86)	0.12
Expressed pleasure at father’s effort to quit	6(4.2)	9(8.0)	1.80(0.38, 8.49)	0.45
Helped father to use substitutes for cigarettes	19(13.4)	1(0.9)	0.07(0.01, 0.59)	0.02

Remark: Values are number (%). For all regression models, odds ratios and regression coefficients were adjusted by age (father, mother & child), father’s education level, years of father smoking, father’s perceived health status, child’s consultation to doctor past month, household income level and number of children at home. Missing data were excluded from analysis.

The proportion of mothers who did not advise the fathers to quit in the past month was similar between pre- and post-legislation (pre: 34.2%, post: 35.9%), but more mothers post-legislation advised 3 times or more (pre: 8.3%, post: 33.1%, p<0.01). There were no significant differences in the proportion of mothers who had action (pre: 23.1%, post: 26.7%, p = 0.62) and gave support to help fathers quit (pre: 28.2%, post: 31.9%, p = 0.88). Slightly more mothers post-legislation placed a ‘no-smoking’ sign at home (pre: 0.7%, post: 6.1%, p = 0.15), advised the fathers to seek professional help (pre: 0.7%, post: 7.8%, p = 0.02), and complimented the fathers when they did not smoke (pre: 7.7%, post: 18.6%, p = 0.02). Fewer mothers helped the fathers use substitutes for cigarettes such as nicotine replacement therapy (pre: 13.4%, post: 0.9%, p = 0.02).

## Discussion

### Summary of results

To our best knowledge, the present study is the first to investigate the impact of smokefree legislation on smoking families with younger children. Our findings did not support the displacement hypothesis that the smokefree legislation would result in increase in smoking in the home, but support the alternative social diffusion hypothesis that the opposite would occur. While there was no substantial change in father’s smoking and quitting, SHS exposure in children post-legislation reduced, which was accompanied by other findings that fewer fathers post-legislation smoked in the presence of children and more mothers took action to protect children from SHS. However, there was no substantial improvement in mothers’ specific advice and action to help the fathers quit smoking after the legislation.

### Interpretation of findings

Based on the report from both fathers and mothers, smoking at home and SHS exposure among children in the home appeared to decrease after the legislation, where was consistent with the reduced hair nicotine level in both children and mothers. These findings are consistent with the hypothesis of social diffusion [15], [23], such that the provision and promotion of smokefree legislation would increase the public approbation on reducing SHS exposure in the indoor environment and encourage more families to create a smokefree home. A study in Scotland supported that such legislation influenced parents to create a smokefree home through increasing their knowledge about the health hazards from SHS and desire to be seen as a caring and socially acceptable model [39]. The displacement hypothesis was not supported, and this is interesting as the present results were observed in a patriarchal society with most smokers being male. In Hong Kong, a large proportion of the population live in densely-populated apartments, and most environments nearby these apartments, especially in public housing estates, were designated as smokefree areas. However, the 2007 smokefree legislation with extensive prohibition of smoking in the outdoor areas was associated with subsequent reduction of fathers’ smoking at home and SHS exposure in their children. Nevertheless, our conclusion is not consistent with another Hong Kong study by Ho etal. (2010), which supported the displacement hypothesis. The inconsistency might be due to the different study design and the children’s age. Ho etal. (2010) included in-school adolescents (equivalent to US grades 2–4, aged 6 to 9), but the present study included much younger children aged 18 months or below. Mothers with younger children might be more concerned about the adverse effect of SHS and more influenced by the legislation, and hence took more action to protect their children from SHS than mothers with older children.

Our finding supported that more families post-legislation created a smokefree home, which might lead to the reduced SHS exposure in children in the home. This is consistent with another finding that the hair nicotine level in mothers’ and children’s hair post-legislation was lower than pre-legislation. Due to the warmer temperature and thicker walls in houses in Hong Kong [35], the air nicotine level in the home was nearly undetectable and thus we had limited biochemical evidence to support the change of smoking behavior at home. However, over 60% of the fathers post-legislation reported that they still smoked at home while their children were not there. Increasing studies showed that tobacco smoke can stick to indoor surfaces and release later as “third-hand smoke” [40], [41]. Third-hand smoke can accumulate in smokers’ home and pose additional health hazards through dermal exposure and inhalation [42]. This means that children and others can still be exposed to the hazards of smoking even if the smokers do not smoke near them. In order to achieve zero exposure to SHS and third-hand smoke in the home, a smokefree home should be advocated.

Another important impact of the smokefree legislation, which had not been explored in previous studies, was the increase in maternal action to protect children from SHS and advice to fathers to quit smoking. Several studies showed that Chinese married women were less likely to attempt to change the smoking behavior of their husbands because Chinese men in general have a more dominant role in the family [32], [33]. Our findings showed that the differences in mother’s action to protect children from SHS exposure between pre- and post-legislation were moderate, including taking the children away from SHS, placing no-smoking signs at home, and increasing the intensity of advising the fathers to quit. However, about 35% of the mothers pre- and post-legislation still did not advise the fathers to quit. Few mothers post-legislation took specific action and showed tangible support to help the fathers quit, such as seeking professional help and comforting the fathers when craving. The smokefree legislation had raised the awareness of the mothers in protecting the children, but was not strong enough for them to support the fathers to go for cessation. Some other factors such as marital relationship that may confound the relationship between the legislation and the mother’s action need further exploration. Our findings support that more specific and effective interventions are needed to motivate and empower mothers with better skills and knowledge to help smoking fathers quit.

### Policy implication

Publicity campaigns and policy implementation were shown to be effective in promoting smoking cessation and utilization of cessation service, especially in the context of the smokefree legislation [11], [12]. However, the lack of comprehensive and persistent cessation campaigns and insufficient funding for cessation services with the implementation of smokefree legislation in Hong Kong were major limitations. According to the budget of Department of Health, the budget for law enforcement increased from US$0.9 million (US$1 = HK$7.8) in 2006 (pre-legislation) to US$3 million in 2007 (post-legislation) [43]. The budget for publicity work of Hong Kong Council for Smoking and Health (COSH) increased from US$0.4 million in 2005 (pre-legislation) to US$1.1 million in 2007 (post-legislation) [44], [45]. Much more resources were allocated to publicity and enforcement of the smokefree legislation (e.g. manpower for patrol and prosecution work). However, smoking cessation services were extended in January 2009, which was two years after the implementation of the new legislation. Legislation can have stronger effect to increase quit attempts, if it is implemented together with early and massive social marketing campaigns for smoking cessation, specific interventions for families and available cessation services.

### Limitations

The strength of this study was the specific focus on the smoking families with younger children. Also, both self-reported smoking status at home and direct measurement of nicotine level were analyzed. MCHCs and SHSCs were selected as the recruitment sites because about 75% of the new born babies, 95% of the primary school students and 80% of the secondary school students in Hong Kong received the free health services in MCHCs and SHSCs, respectively [46], [47]. However, due to the limited resources and permission from Department of Health, only 4 of 32 MCHCs and 2 of 12 SHSCs were selected, which might reduce representativeness of the sample.

The present study had several limitations. Firstly, the RCTs in 2005 and 2006 were not specifically designed to evaluate the effect of the smokefree legislation. The demographic characteristics of the pre- and post-legislation samples showed some differences, which could lead to bias for comparison. To increase the comparability, the post-legislation survey had the same recruitment sites and eligibility criteria with the previous two RCTs. Also, the effect of legislation in the regression models was examined with the adjustment of the significant demographic variables. Secondly, the present study had a smaller sample size than most of the other population-based studies. Due to the limited number of recruitment sites, we pooled the subjects from MCHCs and SHSCs to maximize the sample size, but this might increase the heterogeneity of the subjects. Thirdly, Hong Kong has been commended for effective and evidence-based tobacco control [48]. It would be difficult to differentiate between the impact of smokefree legislation and other measures in the past, although there was no increase in tobacco tax and no substantial change of tobacco control measures during the study period. Future studies may consider comparing the impact of smokefree legislation with other places with a similar history of tobacco control. Lastly, this study was not a cohort study. Our findings were based on 3 cross-sectional surveys of smoking families and smokers at three time points, hence the causal inference on the differences in the outcomes is limited.

### Conclusion

Our findings showed the additional benefits of the smokefree legislation in Hong Kong, which extended the prohibition of smoking to all indoor workplaces and many outdoor areas, in reducing SHS exposure in younger children in the home and increasing mothers’ action to protect their children from SHS. More effort in increasing cessation services and supporting mothers in helping the fathers to quit together with other strong tobacco control policies is needed.

## Supporting Information

File S1 **Supporting tables. Table S1, All outcomes in the analysis. Table S2, Socio-demographic characteristics of fathers and mothers pre- and post-legislation. Table S3, Socio-demographic characteristics of fathers, mothers and children between 2005 RCT and 2007 survey.**
(DOCX)
