# Supplementary material for: Secondhand Smoke Exposure and Maternal Action to Protect Children from Secondhand Smoke: Pre- and Post-Smokefree Legislation in Hong Kong
Source: PLoS One. 2014 Aug 28;9(8):e105781. doi: 10.1371/journal.pone.0105781 (PMC4148325; doi:10.1371/journal.pone.0105781)
Supplement: File S1 — Supporting tables. Table S1, All outcomes in the analysis. Table S2, Socio-demographic characteristics of fathers and mothers pre- and post-legislation. Table S3, Socio-demographic characteristics of fathers, mothers and children between 2005 RCT and 2007 survey. (DOCX) [file pone.0105781.s001.docx]

Table S1: All outcomes in the analysis

| **Outcome** | **Outcomes** | **Type (number of categories)** | **Comparison** |
| --- | --- | --- | --- |
| Father-reported smoking characteristics  (Table 1) | Mean number of cigarettes daily | Ordinal (4) | 2005+2006 versus Post-legislation |
|  | Number of cigarettes when the smoking was the heaviest | Continuous |  |
|  | Fagerstrom score of nicotine dependence (6-item) | Ordinal (3) |  |
|  | Situations of smoking (10-item) | Binary |  |
| Father-reported quit attempt (Table 1) | Had intentionally reduced smoking ever | Binary | 2005+2006 versus Post-legislation |
|  | Had quit attempt ever | Binary |  |
|  | Stage of readiness | Ordinal (4) |  |
| Direct measures of nicotine level  (Table 2) | Nicotine level in hair of mother and children, and in household air with an air monitor | Continuous | 2006 versus Post-legislation |
| Mother-reported SHS exposure among children at home (Table 3) | Father’s smoking frequency within 10 feet of the child in the past week | Ordinal (5) | 2005 versus Post-legislation |
|  | Father’s smoking frequency at home in the past week | Ordinal (5) |  |
|  | Number of smokers (excl. father) smoked within 10 feet of child in past week | Ordinal (4) |  |
|  | Frequency of SHS at home among children | Ordinal (6) |  |
| Mother-reported action to protect child from SHS exposure (Table 4) | Mothers’ action to protect child from SHS exposure (6-item) | Binary | 2005 versus Post-legislation |
| Mother-reported action to help father quit smoking (Table 4) | Intensity of asking father to quit smoking in past month | Ordinal (5) | 2005 versus Post-legislation |
|  | Content of advice (3-item) | Binary |  |
|  | Mother’s actions to help father to quit (7-item) | Binary |  |
|  | Mother’s support in helping father to quit (7-item) | Binary |  |

^1^Leung, D.Y.P., Chan, S.S.C., Lau, C.P., Wong, V. & Lam, T.H. (2008) An Evaluation of the Psychometric Properties of the Smoking Self-Efficacy Questionnaire (SEQ-12) among Chinese Cardiac Patients Who Smoke. Nicotine & Tobacco Research 10(8): 1311-1318.

^2^Ward, R.M., Velicer, W.F.,Rossi, J.S., Fava, J.L. & Prochaska, J.O. (2004) Factorial invariance and internal consistency for the decisional balance inventory—short form. Addictive Behaviors 29(5): 953-958.

Table S2: Socio-demographic characteristics of fathers and mothers pre- and post-legislation

|  | Pre-legislation  2005-2006  (n = 323) | | Post-legislation 2007-2008  (n = 604) | | Chi-square test / t-test  p-value |
| --- | --- | --- | --- | --- | --- |
|  | n | % | n | % |  |
| **Father** |  | |  | |  |
| Mean age, years (SD) | 38.3 (7.8) | | 39.3 (7.8) | | 0.07 |
| Education level |  |  |  |  | <0.01 |
| No/Primary | 32 | 9.9 | 79 | 13.0 |  |
| F1 to F3 | 56 | 17.4 | 211 | 34.9 |  |
| F4 to F7 | 204 | 63.4 | 243 | 40.2 |  |
| Above F7 | 30 | 9.3 | 71 | 11.8 |  |
| Self-perceived physical health | 231 | 72.4 | 388 | 64.2 | 0.01 |
| Poor | 4 | 1.3 | 10 | 1.7 | <0.01 |
| Normal | 84 | 26.3 | 206 | 34.1 |  |
| Good | 212 | 66.5 | 320 | 53.0 |  |
| Very good | 19 | 6.0 | 68 | 11.3 |  |
| Mean age started to smoke (SD) | 18.29 (4.46) | | 18.18 (4.59) | | 0.72 |
| Mean smoking years (SD) | 19.89 (7.95) | | 22.43 (8.6) | | <0.01 |
| **Mother** | (n = 323) | | (n = 742) | |  |
| Mean age, years (SD) | 33.3 (5.8) | | 36.6 (5.7) | | <0.01 |
| Education level |  |  |  |  | <0.01 |
| No/Primary | 31 | 9.3 | 53 | 7.2 |  |
| F1 to F3 | 50 | 15.0 | 226 | 30.7 |  |
| F4 to F7 | 228 | 68.5 | 383 | 52.0 |  |
| Above F7 | 24 | 7.2 | 75 | 10.2 |  |

Table S3: Socio-demographic characteristics of fathers, mothers and children between 2005 RCT and 2007 survey

|  | Pre-legislation  2005  (n=219) | | Post-legislation 2007  (n=183) | | p-value for Chi-square test / t-test | |
| --- | --- | --- | --- | --- | --- | --- |
| **Father** | n | % | n | % |  |  |
| Mean age, years (SD) | 36.3 (7.4) | | 35.8 (6.86) | | 0.56 |  |
| Education level |  |  |  |  | <0.01 |  |
| No/Primary | 17 | 8.2 | 7 | 4.3 |  |  |
| F1-F3 | 16 | 7.7 | 54 | 33.5 |  |  |
| F4-F7 | 159 | 76.4 | 76 | 47.2 |  |  |
| Above F7 | 16 | 7.7 | 24 | 14.9 |  |  |
| Occupation status |  |  |  |  | 0.94 |  |
| Retired | 0 | 0.0 | 1 | 0.6 |  |  |
| Unemployed | 8 | 3.8 | 4 | 2.5 |  |  |
| Currently employed | 200 | 96.2 | 154 | 96.9 |  |  |
| Self-perceived physical health |  |  |  |  |  |  |
| Poor | 1 | 0.5 | 4 | 2.5 | <0.01 |  |
| Normal | 30 | 14.6 | 38 | 23.8 |  |  |
| Good | 174 | 84.9 | 89 | 55.6 |  |  |
| Very good | 0 | 0.0 | 29 | 18.1 |  |  |
| **Mother** |  |  |  |  |  |  |
| Mean age (SD) | 31.2 (4.9) | | 31.6 (4.9) | | 0.36 |  |
| Education level |  |  |  |  |  |  |
| No/Primary | 20 | 9.2 | 7 | 3.9 | <0.01 |  |
| F1-F3 | 24 | 11.0 | 45 | 25.1 |  |  |
| F4-F7 | 164 | 74.9 | 108 | 60.3 |  |  |
| Above F7 | 11 | 5.0 | 19 | 10.6 |  |  |
| Occupation status |  |  |  |  |  |  |
| Housewife | 149 | 68.0 | 95 | 53.1 | <0.01 |  |
| Currently employed | 70 | 32.0 | 84 | 46.9 |  |  |
| Household income (US$1=HK$7.8) |  |  |  |  |  |  |
| Below HK$10,000 | 30 | 14.0 | 30 | 17.6 | <0.01 |  |
| HK$10K – 20K | 118 | 55.1 | 54 | 31.8 |  |  |
| HK$20K – 30K | 42 | 19.6 | 39 | 22.9 |  |  |
| Above HK$30K | 24 | 11.2 | 47 | 27.6 |  |  |
| Perceived good/very good spousal relationship | 219 | 100.0 | 175 | 97.2 | 0.02 |  |
| **Child** |  |  |  |  |  |  |
| Mean age, months (SD) | 4.7 (4.6) | | 6.6 (5.8) | | <0.01 |  |
| Boy | 112 | 51.1 | 90 | 49.7 | 0.66 |  |
| Is the child the first child? | 107 | 48.9 | 85 | 47.0 | 0.38 |  |
| Perceived good/very good child health | 219 | 100.0 | 175 | 98.3 | 0.06 |  |
| No of hospitalization |  |  |  |  |  |  |
| 0 | 202 | 92.2 | 146 | 81.6 | 0.01 |  |
| 1-3 | 17 | 7.8 | 33 | 18.4 |  |  |
| Medical consulation in past month |  |  |  |  |  |  |
| Did not consult | 196 | 89.5 | 128 | 72.7 | <0.01 |  |
| One | 14 | 6.4 | 35 | 19.9 |  |  |
| More than once | 9 | 4.1 | 13 | 7.4 |  |  |
| Number of children at home |  |  |  |  |  |  |
| 1 | 101 | 46.1 | 27 | 15.1 | <0.01 |  |
| 2 | 94 | 42.9 | 93 | 52.0 |  |  |
| 3 | 18 | 8.2 | 49 | 27.4 |  |  |
| 4 | 6 | 2.7 | 7 | 3.9 |  |  |
| 5 | 0 | 0.0 | 3 | 1.7 |  |  |
